# An Exploratory Study of Itolizumab on the Preservation of Beta Cell Function in Adults with Recent-Onset Type 1 Diabetes

**DOI:** 10.3390/jcm11071789

**Published:** 2022-03-24

**Authors:** Eduardo Cabrera-Rode, Ileana Cubas-Dueñas, Janet Rodríguez-Acosta, Yudith García-García, Yelena Torres-López, Claudia Prieto-Noa, Bárbara M. Vázquez-Izada, Maité Ruíz-Reinoso, Ragmila Echevarría-Valdés, Aimee Álvarez-Álvarez, Emma Domínguez-Alonso, Ana Ibis Conesa-González, Teresa González-Calero, Erick Robles-Torres, Silvia Elena Turcios-Tristá, Elizabeth Senra-Estévez, Patricia Hernández-Casaña, Luis Sarmiento

**Affiliations:** 1 Institute of Endocrinology, University of Medical Sciences of Havana, Zapata and D, Plaza, Havana 10400, Cuba; ileana.cubas77@gmail.com (I.C.-D.); yanetra@infomed.sld.cu (J.R.-A.); ygarciagarcia@infomed.sld.cu (Y.G.-G.); yeli9105@nauta.cu (Y.T.-L.); noacmd@gmail.com (C.P.-N.); barbaravi@infomed.sld.cu (B.M.V.-I.); maiterreinoso@infomed.sld.cu (M.R.-R.); ragmila@infomed.sld.cu (R.E.-V.); aimee.alvarez@infomed.sld.cu (A.Á.-Á.); emmada@infomed.sld.cu (E.D.-A.); ana.conesa@infomed.sld.cu (A.I.C.-G.); teregonzalez@infomed.sld.cu (T.G.-C.); erickrt@infomed.sld.cu (E.R.-T.); silviaelena@infomed.sld.cu (S.E.T.-T.); senraest@infomed.sld.cu (E.S.-E.); 2 Center of Molecular Immunology, 216 Street and 15 Avenue, Atabey, Playa, P.O. Box 16040, Havana 11600, Cuba; patriciahc@cim.sld.cu; 3 Immunovirology Unit, Department of Clinical Sciences, Skåne University Hospital, Lund University, 20502 Malmo, Sweden

**Keywords:** type 1 diabetes, Itolizumab, human trials, C-peptide, insulin

## Abstract

We conducted a phase I-IIa, randomized, monocentric, double-blind, placebo-controlled clinical trial to evaluate the safety and impact of the combination treatment of Itolizumab and insulin on preserving beta cell function in adults with recent-onset type 1 diabetes. Twelve patients were randomly assigned to three treatment groups, each receiving a different Itolizumab dose (0.4/0.8/1.6 mg/kg body weight, respectively) and a placebo group. All patients received concomitant intensive multiple-dose insulin therapy. Endogenous insulin secretion was assessed by the measurement of C-peptide during the mixed-meal tolerance test. No serious adverse events were reported. No changes in the total daily insulin doses, glycated hemoglobin levels, and stimulated C-peptide were observed between the Itolizumab and placebo groups at 52 weeks. A significant decrease in stimulated C-peptide was observed during the follow-up period (*p* = 0.012). One subject treated with 1.6 mg of Itolizumab showed a marked increase in the levels of stimulated C-peptide three years after completion of the trial. Taken together, this is the first study to demonstrate that combination treatment with Itolizumab and insulin is safe in humans and does not affect the residual function of beta cells up to 52 weeks. The findings from our study show preliminary evidence that high doses of Itolizumab could potentially arrest the loss of beta cell function in the long term. Further studies with a longer follow-up and larger numbers of patients are envisaged to assess the effect with high dose Itolizumab.

## 1. Introduction

Type 1 diabetes (T1D) is characterized by the immune-mediated destruction of insulin-producing beta cells in the pancreatic islets, leading to insulin deficiency and hyperglycemia [1,2,3]. Even after the diagnosis of T1D, there is still a proportion of beta cells that are functional, but the predominant inflammation and glucotoxicity affect the beta cell responsiveness [4,5]. Given the impact of the immune system on the disease progression, therapeutic strategies using immune modulation are pivotal for preventing the decline in residual beta cell function and preserving beta cell secretory response after the diagnosis of T1D.

Itolizumab is a humanized monoclonal antibody (MAb) that binds to distal domain 1 of CD6 without interfering with the ALCAM ligand [6,7]. Itolizumab modulates T-lymphocyte activation by reducing the production of proinflammatory cytokines (interferon-γ, IL-6, and tumor necrosis factor-alpha (TNF-α)) [8]. Furthermore, it was shown that Itolizumab does not induce cell proliferation or complement-dependent cytotoxicity [7]. As a result, the modulation of CD6 through therapeutic combinations of MAb T1h (Itolizumab) with conventional insulin therapy could control T cell activation and reduce the prevailing proinflammatory pattern. Thus, silencing the aberrant autoreactive immune responses against pancreatic beta cells would restore the immune tolerance and, ultimately, preserve the functional reserve.

There are previous clinical experiences related to the use of Itolizumab in autoimmune diseases, such as psoriasis and rheumatoid arthritis. Importantly, Itolizumab has been demonstrated to be safe and effective for both disorders, achieving a relevant and long-lasting clinical response [7]. Murine anti-CD6 mAB combined with oral insulin can reverse T1D in the mouse NOD model after the onset of the disease [9]; however, the effect of a humanized anti-CD6 monoclonal antibody combined with insulin on the function of beta cells in T1D patients has not been previously assessed. Therefore, this exploratory study examined the safety and impact of the combination treatment of Itolizumab and insulin on the preservation of beta cell function in adults with recent-onset T1D.

## 2. Materials and Methods

### 2.1. Participants and Study Design

The study was a phase I-IIa, randomized, monocentric, double-blind, placebo-controlled clinical trial in which adults with recent-onset T1D were randomly assigned to receive either different doses (0.4/0.8/1.6 mg/kg body weight) of the humanized monoclonal antibody Itolizumab (anti-CD6), or the infusion of a matching placebo. All patients concurrently received intensive insulin therapy throughout the study. The study was approved on 18 December 2012 by the Institutional Ethics Committee of the National Institute of Endocrinology, Havana, Cuba; committee report number: 160 2083 (1403). All subjects involved in the study gave written informed consent to the treatment and study recruitment. The trial was registered in the National Registry for Clinical Trials with registration number RPCEC00000225 under the WHO’s International Clinical Trials Registry Platform (ICTRP) as described (Itolizumab (T1h) combined with insulin in patients with Type I Diabetes Mellitus: https://rpcec.sld.cu/trials/RPCEC00000225-En, accessed on 22 November 2021).

Patients fulfilling all of the following criteria were included in the study: (i) clinical diagnosis of T1D according to the American Diabetes Association T1D criteria, within 12 weeks of study enrollment [1], (ii) male or female T1D patients under intensive treatment with multiple doses of insulin, during four weeks before inclusion, (iii) peak stimulated C-peptide level ≥ 0.2 pmol/mL following a mixed-meal tolerance test (MMTT), and (iv) results from blood count, liver, and kidney function test within their respective reference range. The key exclusion criteria were: (i) severe malnutrition, (ii) any history of malignancy or severe uncontrolled neurological, respiratory, cardiovascular, digestive, or genitourinary disease, (iii) history or diagnosis of acquired or congenital diseases of the hematopoietic system, current or past HIV infection, hepatitis B and C, bronchial asthma, atopic dermatitis or chronic urticaria, (iv) current use of drugs with hyperglycemic effects (e.g., beta-blockers, angiotensin convertase enzyme inhibitors, nicotinic acid, and interferon), (v) current or prior treatment with high doses of glucocorticoids or other immunosuppressive agents (imuran, azathioprine, methotrexate, cyclophosphamide, cyclosporine A, tacrolimus, mycophenolate mofetil, and intravenous immunoglobulin (>400 mg/kg)), (vi) females who are pregnant or lactating, (vii) history of alcohol and drug use, or significant allergy, and (viii) any condition that, in the opinion of the principal investigator, would interfere with the completion of the trial.

Itolizumab was administered as an intravenous infusion, once a week (dose 0.4 and 0.8 mg/kg/day), or every two weeks (dose 1.6 mg/kg/day) for nine weeks and then every four weeks until 24 weeks of treatment. All patients received insulin up to 28 weeks after the last Itolizumab administration. The patients and all investigators were masked to the identity of the study assignment. One (33.3%) patient in the placebo group, two (66.6%) in the 0.4 mg/kg dose group, one (50%) in the 0.8 mg/kg dose group, and one (33.3%) in the 1.6 mg/kg dose group were followed up to three years after the completion of the trial.

### 2.2. Adverse Events

The evaluation of the Itolizumab-related adverse events (e.g., rashes, pruritus, headache, fever, chills, diarrhea, nausea, dyspepsia) was carried out according to the Common Terminology Criteria for Adverse Events, version 3 (CTCAE).

### 2.3. Physical Examination and Insulin Dose

At each visit, a general and system-based physical examination was performed. The body mass index (BMI) was calculated as weight (kilograms) divided by squared height (meters) [10]. The average daily insulin doses were calculated according to body weight (units/kg) during the three days before the visit to the health center.

### 2.4. Laboratory Measurements

Venous blood samples were collected from all patients at the beginning of the study, at 25 weeks and 52 weeks. Another blood specimen was drawn in 5 patients who were followed up for 36 months after completing the trial. All blood specimens were drawn after 8–12 h overnight fast and tested for glycated hemoglobin (HbA1c), glycemia, lipid concentrations (total cholesterol, triglycerides, high-density lipoprotein cholesterol (HDL-c)), creatinine, uric acid, T1D-associated autoantibodies (islet cell autoantibodies (ICA) and zinc transporter-8 autoantibody (ZnT8A)) and fasting blood C-peptide, and MMTT stimulated C-peptide. The levels of fasting blood glucose concentrations (mmol/L), total cholesterol, triglycerides, HDL-c, creatinine, and uric acid were measured using enzymatic methods in an automatic analyzer (Mindray BS-200E, Nanshan, Guangdong, China). HbA1c was determined using commercial kits using an enzymatic method (Cobas HbA1c Test, Mannheim Baden-Wurttemberg, Germany, REF 06378676 190).

Fasting blood C-peptide and MMTT-stimulated C-peptide were measured by an immunoradiometric assay (IRMA) using a commercial kit (IZOTOP, RK-84CT, Hungary). Each MMTT was performed with Nutrial I (composed of 59.5 g of carbohydrates, 18.7 g of fat, and 19.2 g of protein per 100 g) [11] after 7–10 h overnight fasting and after participants had stopped smoking and insulin medications for at least 12 h [11,12,13,14]. The area under the curve C-peptide (AUC CP) during the MMTT was derived via the trapezoidal method. The serum levels of ICA and ZnT8 autoantibodies were determined using ELISA kits (Biomerica, Inc., Irvine, CA, USA, REF 7010, and ELISARSR ZnT8 Limited, Llanishen, Cardiff, UK).

### 2.5. Evaluations of the Outcome of the Trial

Since blood C-peptide levels during an MMTT is the gold-standard measure of endogenous insulin secretion, the beta cell function derived from the area under the curve C-peptide (AUC CP) response to MMTT after treatment with Itolizumab and insulin was evaluated as a primary outcome. Secondary outcomes included glycemic control (expressed by HbA1c levels), daily insulin doses, BMI, hypoglycemic events, and safety parameters (frequency and severity of clinical adverse events and physical, hematological, and biochemical abnormalities).

### 2.6. Statistical Analysis

All statistical analyses were performed using the Statistical Package for Social Sciences (SPSS) version 21. Descriptive analysis was calculated as per the requirement for each variable (qualitative variables are presented as frequencies and percentages; quantitative variables are reported as mean, median, or SD). The efficacy variables were based on (1) changes in the beta cell function and glycosylated hemoglobin levels from baseline across time, and (2) changes in daily insulin requirements to maintain good metabolic control. Clinical characteristics (BMI, insulin dose) and biochemical results (HbA1c, fasting blood glucose, fasting blood C-peptide, AUC-CP) were compared between the study groups using the Kruskal–Wallis test. The Friedman test was used to compare the differences within each group and the total of patients from baseline, at 25 weeks, and at 52 weeks of the trial. The Wilcoxon signed-rank test was used for comparison at 52 weeks and 25 weeks within the groups. The statistical significance was set at *p* < 0.05 in all analyses.

## 3. Results

**Figure 1:**
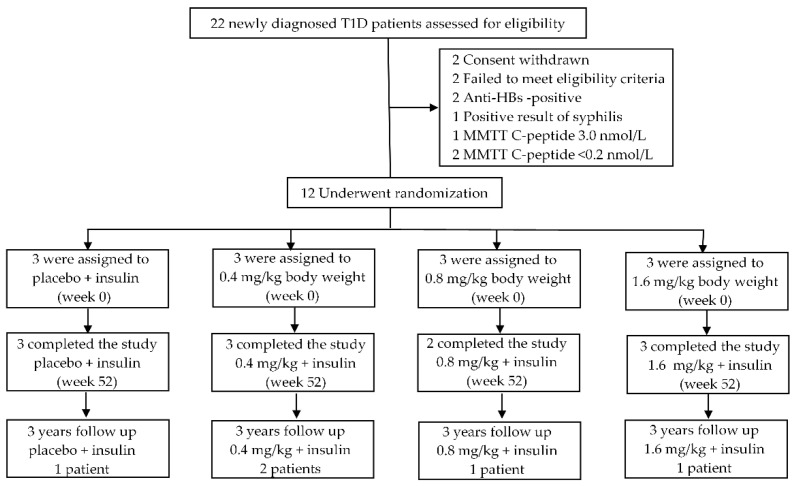
Description of eligible, enrolled, and participating patients in the study. T1D: newly diagnosed T1D patients; anti-HBs: hepatitis B surface antibody; MMTT: mixed-meal tolerance test. The 52-week study period was divided into three stages. During the first stage (week 0–8), Itolizumab was administered through the intravenous route, once a week (dose 0.4 and 0.8 mg/kg/day), or every two weeks (dose 1.6 mg/kg/day). During the second stage (week 9–24), Itolizumab was administered through the intravenous route every four weeks. At the third stage, all patients received insulin up to 28 weeks after the last Itolizumab administration (week 25–52).

A total of 12 newly diagnosed patients with T1D from Havana, Cuba, aged between 19 and 35 (mean age 24.08 ± 4.68) years, underwent randomization to each of the three-dose level groups (0.4/0.8/1.6 mg/kg body weight) or placebo group and 11 (91.6%) completed the study (Figure 1). One participant assigned to the weight-based 0.8 mg/kg dosing withdrew from the study at week 25 after initiation of the trial (Figure 1).

Two of the participants (doses of 0.8 and 1.6 mg/kg, respectively) had a history of diabetic ketoacidosis at diagnosis, but it did not represent a reason for withdrawal or exclusion. The patients enrolled in the study were predominantly male (58.3%, (7/12)), and the frequency of T1D-associated autoantibodies (ICA and/or ZNT8) was 66.7% (8/12). The groups were homogeneous since there were no differences across the groups (placebo and Itolizumab treatment groups) in the variables analyzed at the beginning of the study. The characteristics of the study population and individual information of the participants enrolled in the trial by groups (placebo and Itolizumab treatment groups) at baseline are summarized in Table 1 and Table 2.

During the follow-up period, there were no changes in BMI, daily insulin doses, or glycosylated hemoglobin levels between or within the treatment groups. However, a progressive increase in BMI (median (95% CI)) was observed in all T1D patients, irrespective of their included group. The median baseline was 18.40 (17.83–20.51), and it increased to 21.20 (19.74–21.61) and 20.98 (19.91–21.76) at 25 weeks and 52 weeks, respectively (Friedman test, *p* = 0.035). There were no differences in the fasting blood glucose and C-peptide concentrations between and within the groups during follow-up (data not shown). The area under the curve (AUC-CP) for the C-peptide response to MMTT decreased gradually during the intervention in each treatment group. However, this did not reach statistical significance.

On the other hand, the analysis of the total number of subjects enrolled in the clinical trial showed a significant gradual decrease in AUC-CP during the follow-up period. The AUC-CP (median (95% CI)) baseline was 78.38 (53.21–110.56), and it decreased to 65.85 (55.32–88.84) and 45.90 (36.14–68.13) at 25 weeks and 52 weeks (Friedman test, *p* = 0.012), respectively. Thus, at week 52, there were significant changes in the AUC-CP (*p* = 0.010) (median (95% CI); −21.5 (−59.3–−9.1)) compared to week 25 (−13.1 (−35.8–7.4)). However, by weeks 25 and 52, the treatment and placebo groups showed no significant differences in the mean values of insulin dose, glycosylated hemoglobin, fasting blood glucose, fasting blood C-peptide, BMI, and AUC-CP from the baseline values (data not shown).

**Figure 2:**
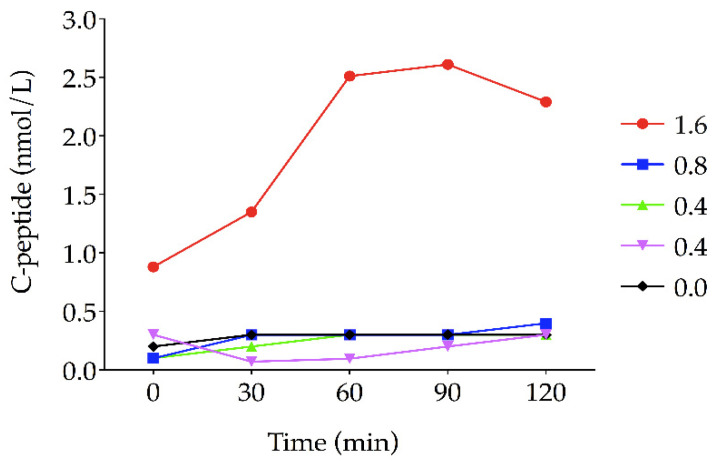
Area under the curve (AUC-CP) for mixed-meal tolerance test (MMTT)-stimulated C-peptide in five patients at three years after completion of the trial.

In the subject treated with 1.6 mg of Itolizumab (islet cell autoantibody-positive subject without ketoacidosis at baseline), the concentrations of C-peptide in the fasting state and during glucose stimulation were three times higher at three years after completion of the trial than those of 52 weeks. In contrast, the rest of the individuals had a pronounced decline in the residual function of the beta cells (Table 3, Figure 2). At three years after completion of the trial, the HbA1c levels were much higher in the subjects treated with doses of 0.4 mg/kg body weight (*n* = 2, 13.7% and 11.9%), 0.8 mg/kg body weight (*n* = 1, 10.3%) and the placebo (*n* = 1, 8.0%), compared to the subject treated with a dose of 1.6 mg/kg body weight (6.1%).

No severe or serious adverse events were observed in the patients receiving Itolizumab during the follow-up period. Fever and chills associated with the first administration of the investigational product appeared in 91.7% (11/12) of the patients. The most frequent adverse reactions were: rash 75% (9), pruritus 75% (9), headache 50% (6), fever 50% (6) and chills 41.7% (5). Most adverse events were peri-infusional (defined as adverse events that occurred within 24 h of infusion), with a decrease in frequency observed after three weeks of treatment. No hematological or infectious adverse events associated with the administration of Itolizumab were reported in any of the subjects involved in the study. Thus, the most adverse events were minor and did not require treatment modification.

## 4. Discussion

This study has provided the first evidence in humans that the combination treatment of Itolizumab and insulin is well tolerated up to the highest tested dose. The findings from our study also provide preliminary evidence that high doses of Itolizumab can potentially arrest the loss of beta cell function in the long term.

In our study, all patients showed decreased C-peptide production and similar exogenous insulin requirement during the post-intervention period. The decline of residual function suggests a progressive loss of the remaining beta cells that were still functional at study entry. As we expected, exogenous insulin therapy at diagnosis of T1D was able to control hyperglycemia. Still, it did not prevent beta cell demise in patients treated with different doses of Itolizumab (0.4/0.8/1.6 mg/kg body weight) or the placebo. The use of intensive therapy with exogenous insulin may also explain the greater weight gain observed in all subjects participating in the study [15,16,17].

The lack of Itolizumab’s effectiveness in arresting the loss of beta cell function at least up to 52 weeks after the first infusion is in sharp contrast with the results obtained using murine anti-CD6 combined with oral insulin in NOD mice. The combination therapy with anti-CD6 and oral insulin markedly reversed recent-onset diabetes in the NOD mouse model. However, the treatment withdrawal led to a decline of the residual beta cell function [9], supporting the role of anti-CD6 in attenuating the decline of beta cell function in newly diabetic NOD mice. Whether different mechanisms of action for anti-CD6 monoclonal antibodies in humans and mice could account for the different results observed needs to be addressed in further studies.

The observed increase in the HbA1c levels in the patients treated with doses of 0.4 mg/kg (*n* = 2, 13.7%, and 11.9%) and 0.8 mg/kg (*n* = 1, 10.3%) compared to placebo (*n* = 1, 8.0%) at three years after the end of the study could be attributed to the difference in age of onset of diabetes in the placebo-treated patient (35 years) and the Itolizumab-treated patients (22 and 23 years). Cumulative evidence suggests that patients diagnosed with T1D early in life generally have a high glycemic load, with substantial differences in HbA1c trajectories based on age at onset [18]. Furthermore, late-onset T1D (after 30 years of age) has been associated with lower levels of HbA1c than early-onset T1D subjects in the first five years of the disease [19].

It is remarkable that a patient assigned to the 1.6 mg dose of Itolizumab increased the ability to produce C-peptide and improved HbA1c levels three years after completion of the trial. In contrast, these variables significantly decreased in those who received doses of 0.4 and 0.8 mg of Itolizumab and placebo and were followed up for three years after the completion of the trial. It is unclear why a high dose of Itolizumab restores the beta cell function in the long term. It is, however, consistent with the findings from other autoimmune diseases, such as rheumatoid arthritis and psoriasis, which revealed a greater overall efficacy of Itolizumab in patients after administering a dose of 1.6 mg [7,20,21,22,23,24]. Notably, the reduced ability of T cells to proliferate and a decreased number of IFN-γ-secreting cells have been reported in patients with psoriasis treated with Itolizumab at the dose of 1.6 mg/kg body weight [7,24,25]. Accordingly, it is tempting to speculate that humanized Itolizumab could have a long-term impact on improving the function of beta cells by protecting the cells from apoptosis caused by proinflammatory cytokines. Therefore, studies of longer duration on a larger number of subjects are necessary to test this possibility.

This study’s major limitation was the small number of participants in each group due to the low incidence of T1D in Havana [26] and the slow pace of patient enrollment. Additionally, only a small number of patients were followed up for a more extended period. Despite these limitations, our study demonstrates that Itolizumab, when administered in combination with insulin, is safe and does not affect the residual function of beta cells up to 52 weeks.

In conclusion, the findings from our study show preliminary evidence of the safety and possible effect of a high dose of Itolizumab combined with insulin on preserving beta cell function in adults with recent-onset T1D. We envisaged the preliminary data of this explorative study priming a broader, appropriately powered long-term follow-up study, in which we will evaluate the long-lasting clinical benefits of this therapeutic combination in patients with recent-onset T1D.

## Figures and Tables

**Table 1:** Baseline characteristics of the study population.

Variable	Newly Diagnosed T1D Patients (*n* = 12)
Male (%)	7 (58.3)
Autoantibodies (ICA and ZnT8A) (%)	8 (66.7)
Age (years)	24.08 ± 4.68
Body mass index (kg/m^2^)	18.40 (CI: 17.83–20.51)
Insulin dose (U/kg)	0.55 (CI: 0.39–0.67)
HbA1c (%)	7.85 (CI: 6.45–9.20)
Fasting blood glucose (mmol/L)	7.05 (CI: 5.83–10.11)
Fasting blood C-peptide (nmol/L)	0.36 (CI: 0.29–0.41)
AUC glucose (mmol × 120 min)	1463.25 (CI: 1178.77–1797.23)
AUC C-peptide (nmol × 120 min)	78.38 (CI: 53.21–110.56)
Total cholesterol (mmol/L)	3.63 ± 0.60
Triglycerides (mmol/L)	0.97 ± 0.26
HDL-c (mmol/L)	1.21 ± 0.17
Creatinine (µmol/L)	64.25 ± 9.43

ICA: islet cell autoantibodies; ZnT8A: zinc transporter-8 autoantibody; HbA1c: glycated hemoglobin; AUC: area under the curve; HDL-c: high-density lipoprotein cholesterol. Data for continuous variables are given as the mean ± SD or the median (95% CI).

**Table 2:** Individual information of the participants enrolled in the trial by groups (placebo and treatment groups) at baseline.

Itolizumab mg/kg + Insulin	Sex	Autoantibodies (ICA and ZnT8A)	Insulin Dosage u/kg	HbA1c%	Fasting C-Peptide	AUC CP
0	F	−	0.6	9.8	0.43	81.2
	M	+ *	0.7	9.7	0.25	47.9
	M	+	0.4	3.2	0.44	92.4
Mean ± SD		*n* = 2	0.6 ± 0.2	7.6 ± 3.8	0.37 ± 0.11	73.8 ± 23.2
0.4	F	+ *	0.80	10.7	0.48	60.0
	M	+ *	0.80	7.9	0.27	50.4
	M	−	0.30	7.8	0.3	193.5
Mean ± SD		*n* = 2	0.6 ± 0.3	8.8 ± 1.6	0.35 ± 0.11	101.3 ± 80.0
0.8	F	−	0.70	8.6	0.42	89.6
	M	+	0.70	9.9	0.24	33.3
	F ^k^	+ *	0.40	7.3	0.41	82.2
Mean ± SD		*n* = 2	0.6 ± 0.2	8.6 ± 1.3	0.36 ± 0.10	68.4 ± 30.6
1.6	M	+ *	0.50	7.0	0.43	136.7
	M	−	0.10	6.9	0.30	75.6
	F ^k^	+	0.40	5.1	0.24	40.05
Mean ± SD		*n* = 2	0.3 ± 0.2	6.3 ± 1.1	0.32 ± 0.10	84.1 ± 48.8

* Analyzed three years after completion of the trial. ^k^ ketoacidosis at diagnosis. AUC CP: area under the curve (AUC-CP) for mixed-meal tolerance test (MMTT)-stimulated C-peptide.

**Table 3:** Levels of MMTT-stimulated C-peptide in five patients during the combination treatment of Itolizumab and insulin and three years after completion of the trial.

Itolizumab Dose + Insulin	MMTT-Stimulated C-Peptide (nmol/L)				
	0 min	30 min	60 min	90 min	120 min
1.6 mg/kg					
Baseline	0.43	1.08	1.4	1.44	0.84
6 months	0.38	0.85	0.85	1.07	0.82
12 months	0.24	1.08	0.91	0.6	0.72
36 months *	0.88	1.35	2.51	2.61	2.29
0.8 mg/kg					
Baseline	0.41	0.60	0.85	0.63	0.91
6 months	0.38	0.38	0.71	0.93	0.85
12 months	0.33	0.30	0.24	0.13	0.30
36 months *	0.10	0.30	0.30	0.30	0.40
0.4 mg/kg					
Baseline	0.48	0.31	0.57	0.59	0.58
6 months	0.39	0.52	0.47	0.63	0.66
12 months	0.38	0.41	0.41	0.74	0.74
36 months *	0.10	0.20	0.30	0.30	0.30
0.4 mg/kg					
Baseline	0.27	0.31	0.55	0.40	0.57
6 months	0.22	0.33	0.41	0.60	0.60
12 months	0.33	0.33	0.30	0.38	0.30
36 months *	0.30	0.07	0.095	0.20	0.30
0.0 mg/kg					
Baseline	0.25	0.36	0.44	0.45	0.44
6 months	0.28	0.30	0.47	0.61	0.69
12 months	0.08	0.11	0.25	0.33	0.30
36 months *	0.20	0.30	0.30	0.30	0.30

MMTT: mixed-meal tolerance test; * three years after completion of the trial.

## Data Availability

The data presented in this study are available upon reasonable request from the corresponding author.
